# Robust neutralization of emerging JN.1 subvariants by the updated JN.1 vaccine: rationale for catch-up booster recommendations in high-risk individuals

**DOI:** 10.3389/fimmu.2025.1696676

**Published:** 2026-01-05

**Authors:** Ju-yeon Choi, Su-Hwan Kim, Soyoung Park, Hye Min Jang, Young Jae Lee, Hye-Jin Kim, Dokeun Kim, Jae-Hoon Ko, Hye-Sook Jeong

**Affiliations:** 1 Division of Clinical Research for Vaccine, Center for Vaccine Research, National Institute of Infectious Diseases, National Institute of Health, Korea Disease Control and Prevention Agency, Cheongju, Republic of Korea; 2 Division of Infectious Diseases, Department of Medicine, Samsung Medical Center, Sungkyunkwan University School of Medicine, Seoul, Republic of Korea

**Keywords:** COVID-19, cross-reactive, JN.1 subvariant, neutralization, SARS-CoV-2

## Abstract

The updated JN.1 monovalent vaccine (MoV) for the 2024–2025 season elicits strong cross-neutralizing activity against subsequently circulating SARS-CoV-2 variants in 2025, including the recently emerging NB.1.8.1, with titer increases comparable to those against the vaccine strain JN.1. The immune response induced by the JN.1 MoV was markedly stronger than that induced by XBB.1.5 MoV in the previous season, both against the vaccine strain and post-vaccine variant (all *P* < 0.001). These results likely reflect improved background immunity to circulating variants and support the use of catch-up booster vaccination during the summer surge, particularly for high-risk groups.

## 1 Introduction

Since the introduction of wild-type (WT) COVID-19 vaccines in 2021, updated formulations targeting emerging variants, including WT-containing bivalent (BiV) and variant-specific monovalent (MoV) vaccines, have been developed. However, concerns have been raised regarding the limitations of annual vaccine updates, considering the persistent antigenic imprinting from the original WT vaccine and the rapid turnover of the dominant variants (1–3). Although the XBB.1.5 MoV was introduced during the 2023–2024 vaccination season, the JN.1 variant rapidly became the globally dominant strain that winter and remains in circulation as a variant of interest (VOI) (4). After the rollout of the JN.1 MoV for the 2024–2025 season, several JN.1 subvariants, including KP.3, KP.3.1.1, XEC, LP.8.1, and NB.1.8.1, have emerged and are presently classified as variants under monitoring (VUMs) (**Supplementary Figures S1, S2**) (5). Recently, NB.1.8.1 has been rapidly spreading worldwide (6), and has been reported as the predominant circulating strain during the summer surge in Republic of Korea (**Supplementary Figure S3**). In response, the Korea Disease Control and Prevention Agency (KDCA) actively recommended catch-up vaccination with the JN.1 MoV for unvaccinated individuals in high-risk groups and extended the free provision to individuals aged ≥ 65 years and those with immunocompromised conditions. To provide immunologic background for catch-up vaccination during the 2025 summer surge dominated by JN.1 sublineages, we evaluated the immunogenicity of the JN.1 MoV compared with that of the previous XBB.1.5 MoV, and analyzed its cross-neutralizing activity against the subsequently circulating VUMs.

## 2 Material and methods

### 2.1 Study participants and serum collection

Serum samples were collected from two independently established cohorts of healthcare workers (HCWs), each designed to assess the immune response to either XBB.1.5 or JN.1 MoV (7, 8). The JN.1 MoV cohort recruited individuals aged ≥ 65 years, whereas the XBB.1.5 cohort was limited to adults aged < 65 years because of retirement. Each cohort originally consisted of 60 and 120 participants, respectively, and a subset of participants who underwent plaque reduction neutralization test (PRNT) was included in the analyses. While some PRNT data for the XBB.1.5 MoV cohort have been reported in a previous publication (7), the data for the JN.1 MoV cohort and a comparison with the XBB.1.5 cohort are novel to the present study. This study was reviewed and approved by the Institutional Review Board of the Korea National Institute of Health (approval no. 2023-02-03-2C-A) and Samsung Medical Center (approval no. 2021-01-165). All participants provided written informed consent prior to sample collection. The study was conducted in accordance with the principles of the Declaration of Helsinki and national ethical guidelines.

### 2.2 Virus isolates

Authentic SARS-CoV-2 isolates, KP.3 (NCCP no.43496), XEC (NCCP no.43511), LP.8.1.1 (NCCP no.43512), NB.1.8.1 (NCCP no.43513) and JN.1(NCCP no.43488), were obtained from the National Culture Collection of Pathogens (Cheongju, Republic of Korea). These isolates were originally collected and the whole genome was sequenced by the Division of Emerging Infectious Diseases, KDCA, and was propagated once in Vero E6 cells before use in neutralization assays.

### 2.3 Cell culture

Vero E6 cells (Vero 76, ATCC CRL-1586™) were maintained in Dulbecco’s modified Eagle’s medium (high glucose) supplemented with 10% fetal bovine serum (FBS; Gibco), 1× penicillin-streptomycin (Gibco), and 1 µg mL^-^¹ puromycin at 37°C with 5% carbon dioxide (CO_2_).

### 2.4 Binding antibody assays

Anti–spike protein antibodies (Sab) were measured using the Elecsys^®^ Anti–SARS-CoV-2 S assay (Roche Diagnostics) on cobas e analyzers employing the electrochemiluminescence immunoassay (ECLIA) method. A Sab level ≥ 0.8 U/mL was considered positive (linear range 0.4–250 U/mL). Automated on-board dilution was performed up to 1:50, with additional manual dilution to 1:200 for saturated samples (3). The assay’s U/mL values are consistent with the WHO BAU/mL standard and show strong linear correlation with neutralizing antibody titers (9–12). Anti–nucleocapsid protein antibodies (Nab) were assessed using the Elecsys^®^ Anti–SARS-CoV-2 assay (Roche Diagnostics, ECLIA method). A result ≥1.0 cut-off index was defined as positive, consistent with its validated high sensitivity and specificity for detecting prior SARS-CoV-2 infection (13).

### 2.5 PRNT

Neutralizing antibody titers against SARS-CoV-2 variants were measured using the 50% PRNT (PRNT_50_). Authentic SARS-CoV-2 isolates (JN.1, KP.3, XEC, LP.8.1.1 and NB.1.8.1) were used in this study. Serial two-fold dilutions of heat-inactivated serum samples were mixed 1:1 with virus suspensions containing 40 plaque-forming unit (PFU) per well and incubated at 37°C for 1 h. The serum–virus mixtures were subsequently added to Vero E6 cell monolayers pre-seeded in 12-well plates and incubated at 37°C for 1 h to allow virus adsorption. After adsorption, 0.75% agarose overlay medium was added to each well. Plates were incubated for 2 d at 37°C with 5% CO_2_, after which the cells were fixed with 4% paraformaldehyde and stained with 0.1% crystal violet to visualize the plaques. The plaques were manually counted. Neutralizing titers were expressed as ND_50_, the reciprocal serum dilution that led to a 50% reduction in plaque counts compared with that in the virus-only controls. Furthermore, the ND_50_ values were calculated using the Spearman–Karber formula. Samples with ND_50_ ≥ 20 were considered seropositive. Samples below the detection limit (ND_50_ < 10) were assigned a value of 5 for statistical analysis.

### 2.6 Statistical analysis

We compared the baseline characteristics and laboratory test results using the Student’s *t*-test or Mann–Whitney *U* test for continuous variables and the chi-square test for categorical variables. The Wilcoxon matched-pair signed-rank test was used for paired comparisons. The Kruskal–Wallis test, followed by Dunn’s multiple comparison test, was used for comparisons among three or more groups. All *P* values were two-tailed, and statistical significance was defined as *P* < 0.05. All analyzes were performed using GraphPad Prism (version 10.5.0).

## 3 Results

### 3.1 Neutralizing activity against the vaccine strain and post-vaccine variant of the XBB.1.5 and JN.1 MoVs

First, to compare background immunity and MoV effects between the 2023–2024 and 2024–2025 seasons, we assessed the neutralizing activity against the vaccine strain (XBB.1.5 for the XBB.1.5 MoV and JN.1 for JN.1 MoV) and the immediately subsequent variants (post-vaccine strain; JN.1 for XBB.1.5 MoV and KP.3 for JN.1 MoV). Paired pre- and post-vaccination sera were obtained from 40 HCWs in the XBB.1.5 MoV cohort and 37 HCWs in the JN.1 MoV cohort and analyzed using the PRNT (7, 8). The baseline characteristics were similar between the groups except for age, with the individuals in the JN.1 cohort being older (**Table 1**). Both cohorts exhibited robust immune responses following vaccination, as indicated by the Sab levels (**Supplementary Figure S4A**), PRNT titers against vaccine strains (**Supplementary Figure S4B**), and PRNT titers against post-vaccine variants (**Supplementary Figure S4C**; all *P* < 0.001). The PRNT ratio of the post-vaccination variant to the vaccine strain did not significantly alter following vaccination (**Supplementary Figure S4D**).

**Table 1:** Baseline characteristics of the cohort participants.

Variables	XBB.1.5 MoV n = 40	JN.1 MoV n = 37	*P* value
Demographics			
Age, years	44.2 ± 8.0	50.8 ± 9.5	0.001
Male	14 (35.0)	14 (37.8)	0.796
BSA, m^2^	1.7 ± 0.2	1.7 ± 0.2	0.939
Underlying diseases			
Hypertension	1 (2.5)	3 (8.1)	0.346
Diabetes mellitus	0 (0.0)	2 (5.4)	0.228
Thyroid cancer, treated	1 (2.5)	0 (0.0)	1.000
Antigenic stimulation histories			
Primary vaccine series			
AdV-AdV	26 (65.0)	25 (67.6)	0.674
mRNA-mRNA	7 (17.5)	8 (21.6)	
AdV-mRNA	7 (17.5)	4 (10.8)	
WT mRNA booster (3^rd^ dose)	40 (100.0)	36 (97.3)^*^	0.481
WT+BA.4/5 mRNA BiV (4^th^ dose)	20 (50.0)	25 (67.6)	0.118
XBB.1.5 MoV (5^th^ dose)	NA	18 (48.6)	NA
Pre-vaccination Nab			
Titer, COI	72.5 (7.3–187.0)	98.2 (30.9–189.2)	0.528
Positive	40 (100.0)	37 (100.0)	1.000

Data are presented as the number (percent), mean ± standard deviation, or median (interquartile range). ^*^One participant received a protein subunit vaccine as the 3^rd^ dose.

AdV, adenovirus-vector vaccine; BiV, bivalent vaccine; BSA, body surface area; COI, cut-off index; MoV, monovalent vaccine; mRNA, messenger ribonucleic acid vaccine; Nab, anti-nucleocapsid antibody; NA, not applicable; WT, wild-type.

**Figure 1:**
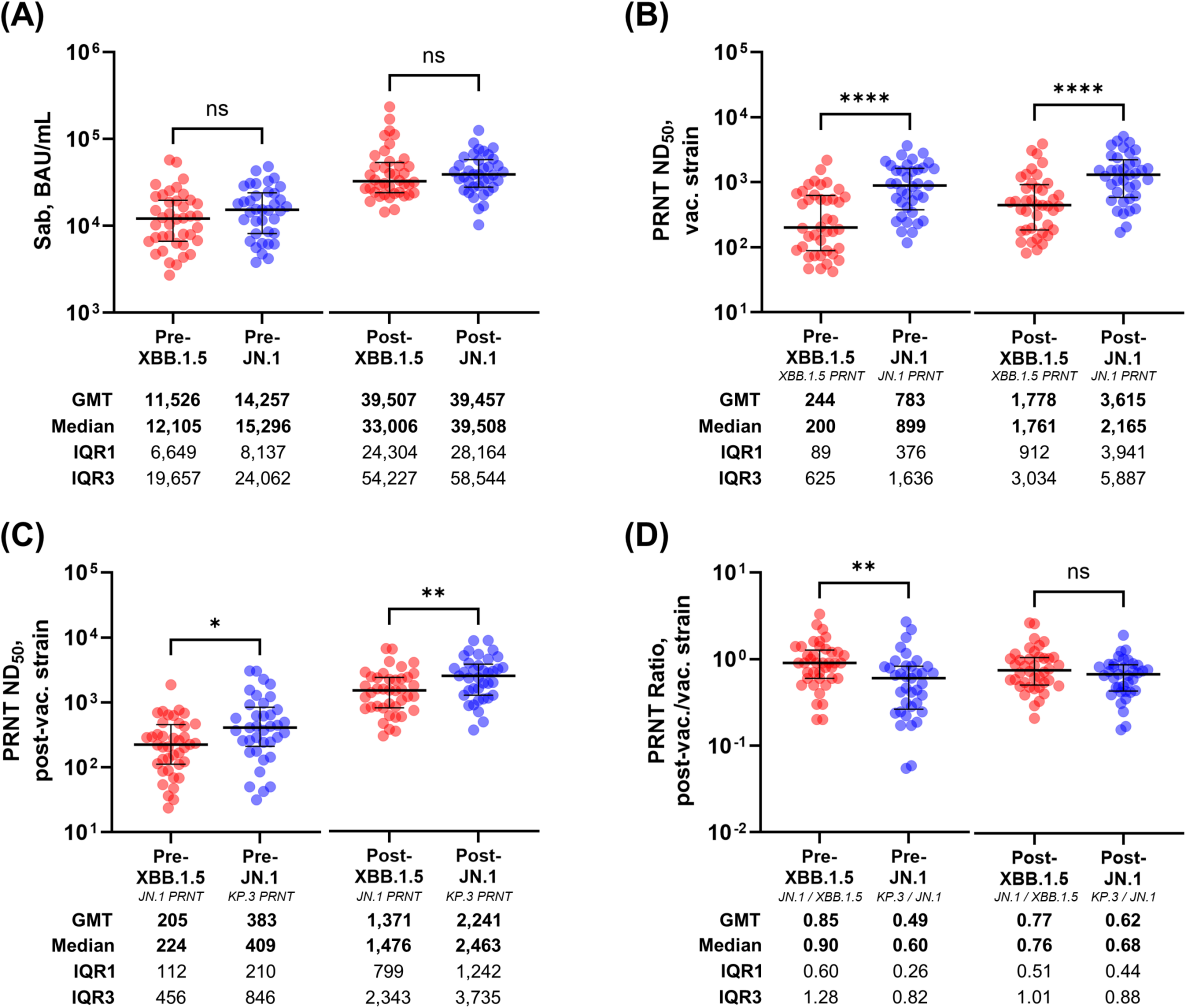
Comparison of the pre- and post-vaccination antibody titers in the recipients of the XBB.1.5 and JN.1 MoVs. Pre- and post-vaccination comparisons are illustrated for the Sab levels **(A)**, PRNT titers against the vaccine strains **(B)**, PRNT titers against the post-vaccine variants **(C)**, and the PRNT ratio of the post-vaccine variant to the vaccine strain **(D)**. *P* < 0.05 (*), *P* < 0.01 (**), *P* < 0.001 (***); and *P* < 0.0001 (****). MoV, monovalent vaccine; Sab, anti-spike antibody; PRNT, plaque-reduction neutralization test; ND_50_, 50% neutralization dose; GMT, geometric mean titer; IQR, interquartile range; GM, geometric mean.

Notably, post-vaccination PRNT titers were significantly higher in the JN.1 cohort than in the XBB.1.5 cohort for the vaccine strain and post-vaccine variant (both *P* < 0.001) despite similar Sab levels (**Figures 1A–C**). Additionally, pre-vaccination PRNT titers against both strains were markedly higher in the JN.1 cohort, suggesting an elevated baseline immunity level likely owing to previous XBB.1.5 vaccination and natural exposure (7). Although the PRNT ratios were lower at the baseline in the JN.1 group, they were comparable between the cohorts following vaccination (**Figure 1D**). Taken together, the JN.1 vaccine induced markedly stronger neutralizing activity against the vaccine strain and post-vaccine variants than the XBB.1.5 vaccine, likely owing to elevated background immunity to circulating variants.

### 3.2 Neutralizing activity against emerging SARS-CoV-2 JN.1 subvariants

Next, we evaluated the cross-neutralization capability of the JN.1 MoV against the circulating variants in 2025. As of 12 July 2025, JN.1 is classified as a VOI, and its subvariants, including KP.3, KP.3.1.1, XEC, LP.8.1, NB.1.8.1 and XFG are classified as VUM (14). Among these, we conducted PRNT assays against KP.3, XEC, LP.8.1.1 and NB.1.8.1 using serum samples from 20 individuals vaccinated with the JN.1 MoV. This group included 10 HCWs in the JN.1 MoV cohort and 10 additional healthy volunteers aged ≥ 65 years (**Supplementary Table S1**).

**Figure 2:**
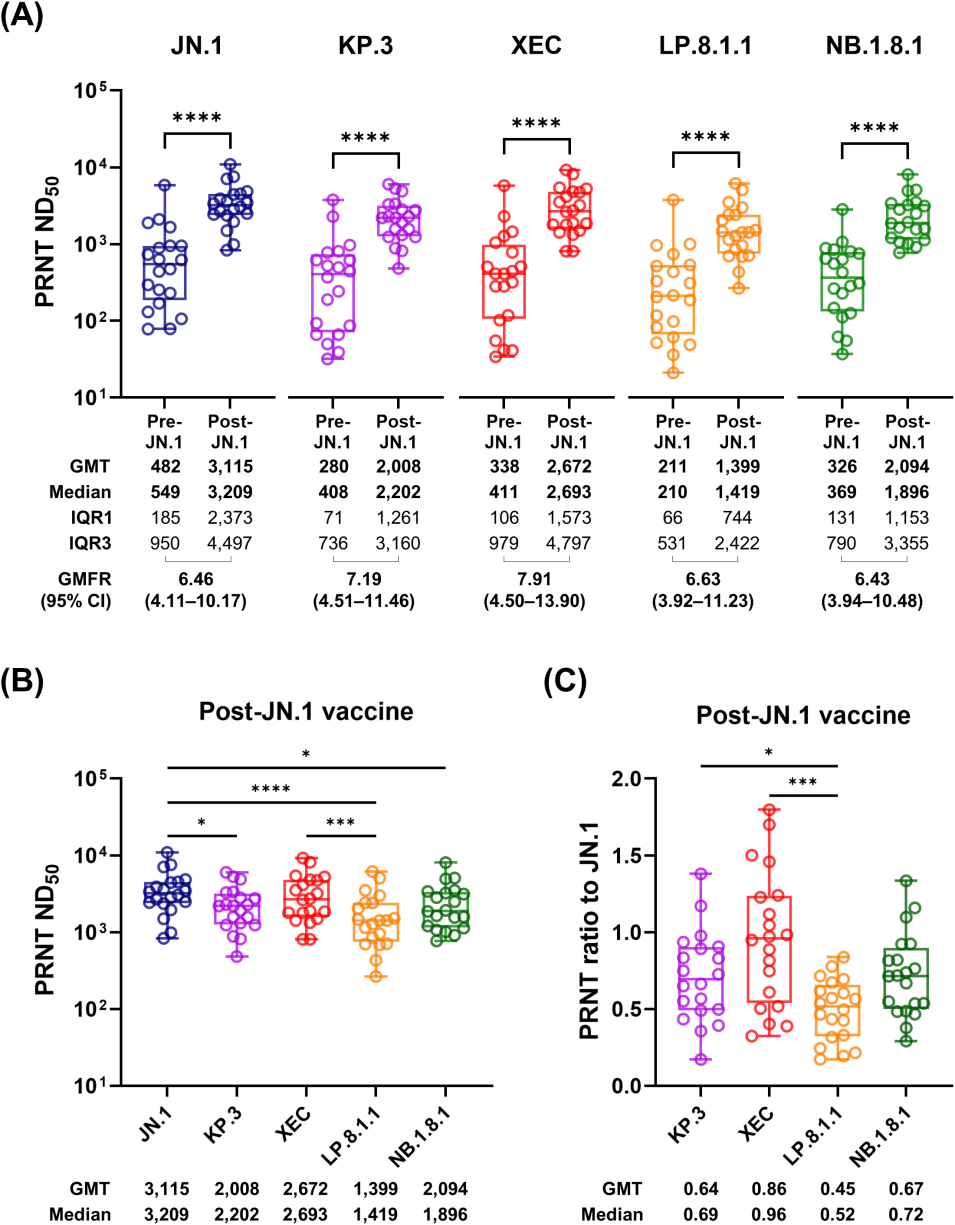
neutralization activity against the recently circulating VUMs in the recipients of the JN.1 MoV. Pre- and post-vaccination comparisons of the PRNT titers against JN.1, KP.3, XEC, LP.8.1.1 and NB.1.8.1 are indicated in **(A)**. Post-vaccination PRTN titers for each variant **(B)** and PRNT ratios relative to JN.1 **(C)** are also presented. *P* < 0.05 (*), *P* < 0.01 (**), *P* < 0.001 (***); and *P* < 0.0001 (****). VUM, variant under monitoring; MoV, monovalent vaccine; PRNT, plaque-reduction neutralization test; ND_50_, 50% neutralization dose; GMT, geometric mean titer; IQR, interquartile range; GM, geometric mean.

Notably, the JN.1 MoV induced a significant elevation in the PRNT titers against all tested VUMs (all *P* < 0.005; **Figure 2A**), with robust fold increases: a geometric mean fold rise (GMFR) of 6.46 for JN.1 (95% CI 4.11–10.17), 7.19 for KP.3 (95% CI 4.51–11.46), 7.91 for XEC (95% CI 4.50–13.90), 6.63 for LP.8.1.1 (95% CI 3.92–11.23) and 6.43 for NB.1.8.1 (95% CI 3.94–10.48). Post-vaccination PRNT titers were highest against JN.1 and were generally comparable across the VUMs, except for LP.8.1.1 (**Figures 2B, C**). These titers were approximately 10- to 25-fold higher than the previously estimated 50% protective threshold (ND_50_ 118.25), which was originally derived for the WT vaccine and virus (15). This suggests that the 2024–2025 JN.1 MoV booster may elicit robust immunity against the VUMs that subsequently circulated in 2025.

## 4 Discussion

After the World Health Organization (WHO) declared in May 2023 that COVID-19 no longer constituted a Public Health Emergency of International Concern, the pandemic ultimately transitioned to the endemic phase. Most countries, including South Korea, shifted from total case counting to sentinel surveillance (16). At present, global trends in the SARS-CoV-2 variants largely rely on the reports from GISAID, which provide the relative genome frequency of each variant by region over time (5). The XBB.1.5 variant, the vaccine strain for the 2023–2024 season, peaked in April 2023, followed by JN.1’s emergence, which dominated the first half of 2024 and continued to be detected across several continents (**Supplementary Figure S1**). Consequently, JN.1 was selected as the vaccine strain for the 2024–2025 season, and its subvariants have since circulated globally (**Supplementary Figure S2**). The JN.1 sublineage NB.1.8.1 had increased in global prevalence by the end of May, accounting for 21% of the submitted sequences, up from 9% just one month earlier (17). This increase was especially observed in East Asia, including China, Hong Kong, and South Korea. LP.8.1, the most recent dominant circulating lineage, peaked globally in April 2025 but now appears to be gradually displaced by NB.1.8.1 in multiple regions (**Supplementary Figure S2**).

The advisory groups of the WHO, FDA and EMA have recommended considering JN.1 or its sublineages as the vaccine strain for the 2025–2026 season (18–20); however, real-world neutralization data against the most recently circulating variants in the JN.1-vaccinated individuals remain limited. Herein, we found that the JN.1 MoV exhibited broad cross-neutralization activity against the circulating VUMs, especially the recently emerging NB.1.8.1. Notably, this robust neutralizing activity reflects the fair cross-activity of the JN.1 MoV and the cumulative effect of previous XBB.1.5 MoV and natural exposures. PRNT ND_50_ titers against the vaccine strain and post-vaccine variants were markedly higher than those induced by XBB.1.5 MoV, and titers against the most recent VUMs were comparable with each other. These titers were approximately 10- to 25-fold higher than the estimated 50% protective level derived from the WT vaccine and virus. These results provide timely evidence to support catch-up booster vaccination in high-risk populations and to guide vaccine strain selection for the 2025–2026 season. High-risk individuals remain vulnerable to infection as non-pharmaceutical interventions, such as mask-wearing and social distancing, have largely been discontinued, despite population-level immunity against circulating SARS-CoV-2 variants increasing since the transition to the endemic phase. Nevertheless, the booster vaccine coverage during the 2024–2025 season among the elderly remains low worldwide: 27.8% among adults aged ≥ 65 years in the United States (21), a median of 7.4% among those aged ≥ 60 years and 10.8% among those aged ≥ 80 years in Europe (22) and 47.4% among adults ≥ 65 years in South Korea (23). Since recent COVID-19 surges are not limited to winter season (24, 25), continued public recommendations for catch-up booster vaccination are necessary, supported by evidence of vaccine effectiveness and safety.

When compared with the XBB.1.5 MoV administered in the 2023–2024 season, the JN.1 MoV cohort exhibited higher PRNT titers against both the vaccine strain and the post-vaccine circulating strains at both the pre- and post-vaccination time points. Although clinical factors that may influence vaccine immunogenicity—such as age and prior vaccination history—were not fully controlled, baseline characteristics were largely comparable between the two groups except for age, which was higher in the JN.1 cohort. Therefore, the observed difference may reflect the maturation of immunity against circulating SARS-CoV-2 variants, acquired through vaccination and/or natural boosting during the intervening year. Ideally, maintaining the same participants across the two cohorts would have enabled a more direct comparison of the vaccines. However, because vaccination decisions depended on individual preference, maintaining the same participants across both seasons was not feasible, and this inevitably constrained the direct comparability of the two vaccine cohorts.

This study has some limitations. First, the number of serum samples that underwent PRNT against recently circulating VUMS was limited. However, the robust cross-activity noted was consistent with the comparative data from the XBB.1.5 and JN.1 MoV cohorts against the post-vaccine variant. Second, we assessed only the humoral immune response. Nonetheless, CD4^+^ and CD8^+^ T-cell epitopes across the Omicron lineages, including JN.1 derivatives, remain largely conserved (26), and *de novo* memory T-cell responses to circulating strains have been demonstrated to develop (27). Third, we used the Elecsys^®^ kits, which were developed based on the RBD of WT SARS-CoV-2, to assess binding antibody response after recently updated vaccines. We have continuously evaluated the correlation between Sab levels measured by the Elecsys^®^ kits and PRNT titers against circulating strains (11, 12, 28, 29). Although the correlation coefficients varied by variant, linear correlations were consistently observed. We also tested several variant-specific binding antibody assays, but their performance did not surpass that of the Elecsys^®^ assay (29). In addition to our previous findings, other groups have also reported that Sab titers from Elecsys^®^ assay moderately correlate with neutralizing activity against Omicron subvariants (30). Therefore, Sab titers can be interpreted as supportive indicators of the overall antibody response, whereas direct neutralizing activity against each variant should be inferred from PRNT titers.

In conclusion, the 2024–2025 JN.1 MoV elicited robust cross-reactive neutralizing activities against subsequently circulating VUMs in 2025, including the recently emerging NB.1.8.1, supporting the use of catch-up booster vaccination during the summer surge. The immune response induced by the JN.1 MoV was markedly stronger than that induced by the XBB.1.5 MoV of the previous season, likely reflecting improved background immunity to circulating variants.

## Data Availability

The original contributions presented in the study are included in the article/**Supplementary Material**. Further inquiries can be directed to the corresponding authors.
