# Supplementary material for: Robust neutralization of emerging JN.1 subvariants by the updated JN.1 vaccine: rationale for catch-up booster recommendations in high-risk individuals
Source: Front Immunol. 2026 Jan 5;16:1696676. doi: 10.3389/fimmu.2025.1696676 (PMC12812961; doi:10.3389/fimmu.2025.1696676)
Supplement: Supplementary file 1 [file Table1.docx]

Supplementary Material

# Supplementary Figures and Tables

## Supplementary Table

**Supplementary Table 1. Baseline characteristics of the cohort participants who underwent PRNT against the JN.1 sublineages**

| **Variables** | **Participants who underwent PRNT against JN.1 sublineages,** n = 20 |
| --- | --- |
| **Demographics** |  |
| Age, years | 58.4 ± 12.1 |
| Male | 9 (45.0) |
| BSA, m^2^ | 1.7 ± 0.2 |
| **Underlying diseases** |  |
| Hypertension | 4 (20.0) |
| Diabetes mellitus | 5 (25.0) |
| Dyslipidemia | 6 (30.0) |
| Hypothyroidism | 1 (5.0) |
| **Antigenic stimulation histories** |  |
| Primary vaccine series |  |
| AdV-AdV | 15 (75.0) |
| mRNA-mRNA | 4 (20.0) |
| AdV-mRNA | 1 (5.0) |
| WT mRNA booster (3^rd^ dose) | 20 (100.0) |
| WT+BA.4/5 mRNA BiV (4^th^ dose) | 15 (75.0) |
| XBB.1.5 MoV (5^th^ dose) | 10 (50.0) |
| **Pre-vaccination Nab** |  |
| Titer, COI | 106.1 (29.2–182.3) |
| Positive | 20 (100.0) |

Data are presented as number (percent), mean ± standard deviation or median (interquartile range).

Abbreviations: AdV, adenovirus vector vaccine; BiV, bivalent vaccine; BSA, body surface area; COI, cutoff index; MoV, monovalent vaccine; mRNA, messenger ribonucleic acid vaccine; Nab, anti-nucleocapsid antibody; WT, wild type

## Supplementary Figures


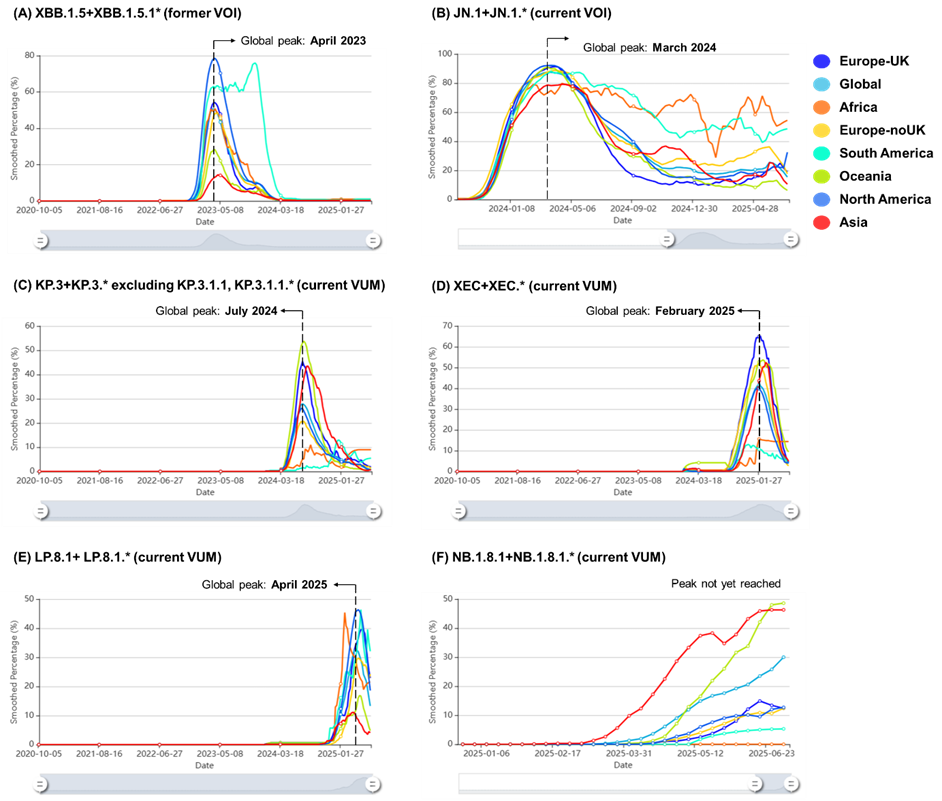


**Supplementary Figure 1. Global trends of the SARS-CoV-2 variants investigated in the present study.** Trends were captured from the GISAID webpage (https://gisaid.org/hcov19-variants/) on 10 July 2025 and are presented as the relative genome frequency of each variant by region over time. The vaccine strain of the XBB.1.5 MoV peaked globally in April 2023 and nearly disappeared by March 2024 (A). JN.1, a post-vaccine variant of XBB.1.5 MoV peaked in March 2024 and continues to be detected globally (B). KP.3 emerged as a post-vaccine variant of the JN.1 MoV and peaked worldwide in July 2024 (C). The XEC revealed a global surge in the second half of 2024, peaking in February 2025 (D). LP.8.1 emerged in late 2024, peaked globally in April 2025 and remained in circulation (E). NB.1.8.1 has shown a continuous rise since early 2025, with peaks yet to be observed (F).

Abbreviations: SARS-CoV-2, severe acute respiratory syndrome coronavirus 2; GISAID, Global Initiative on Sharing All Influenza Data; MoV, monovalent vaccine


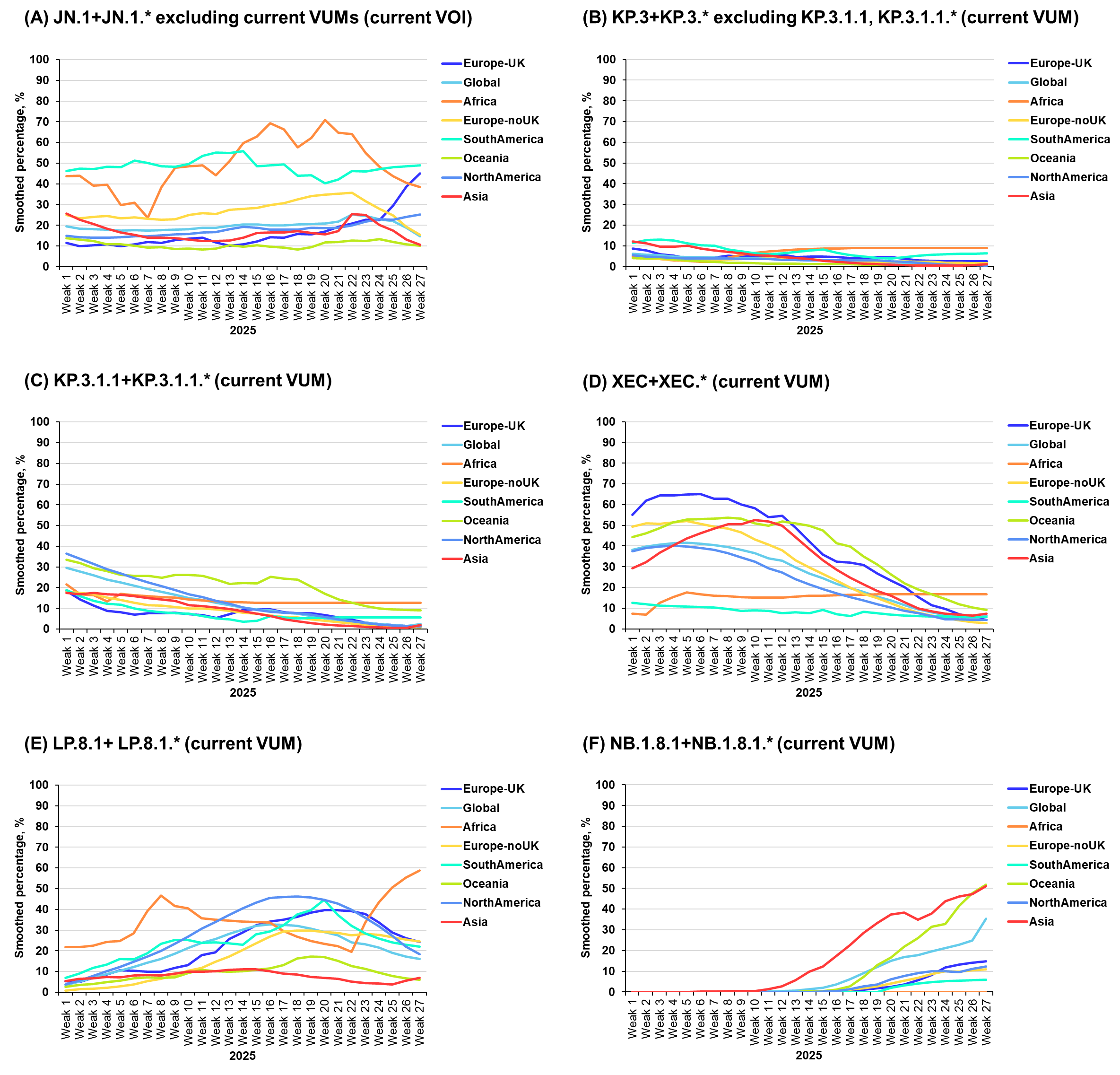


**Supplementary Figure 2.** **Global trends of JN.1 (VOI) and its subvariants designated as VUMs in 2025.** Trend data were extracted from the GISAID webpage (https://gisaid.org/hcov19-variants/) as of 10 July 2025 and are presented as the relative genome frequency of each variant by region during 2025. JN.1 has been reported at a steady proportion across continents (A), whereas KP.3 has been detected at an extremely low frequency (B). KP.3.1 (C) and XEC (D) demonstrated distinct declining trends, whereas LP.8.1 (E) and NB.1.8.1 (F) recently became predominant.

Abbreviations: VOI, variant of interest; VUM, variant of monitoring; GISAID, Global Initiative on Sharing Influenza Data


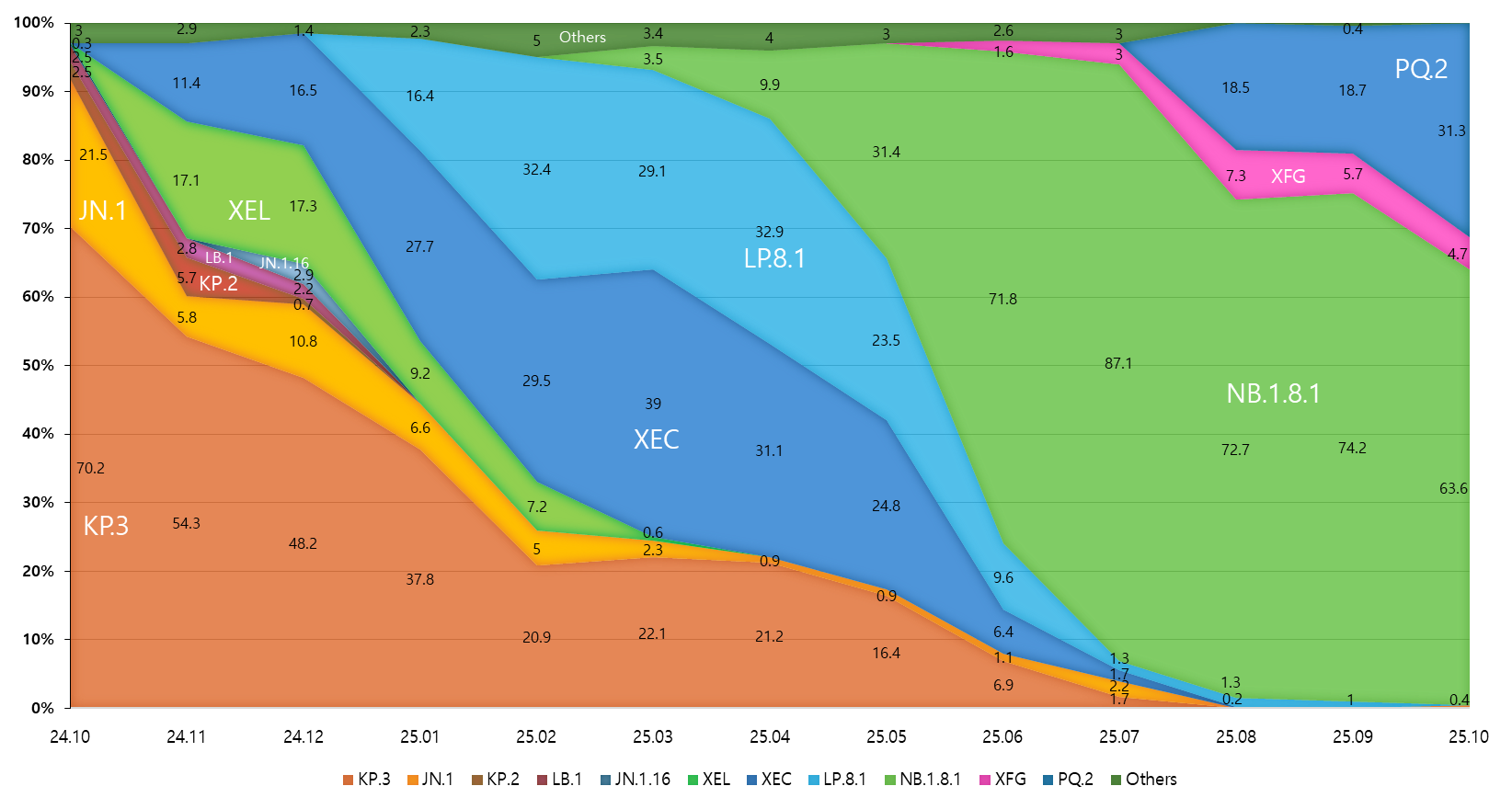


**Supplementary Figure 3.** **Domestic trend of SARS-CoV-2 variants detected in Korea from October 2024 to October 2025.** Trend data were extracted from the KDCA reports as of November 2025 and are presented as monthly proportion each variant.

Abbreviations: SARS-CoV-2, severe acute respiratory distress syndrome coronavirus 2; KDCA, Korea disease control and prevention agency


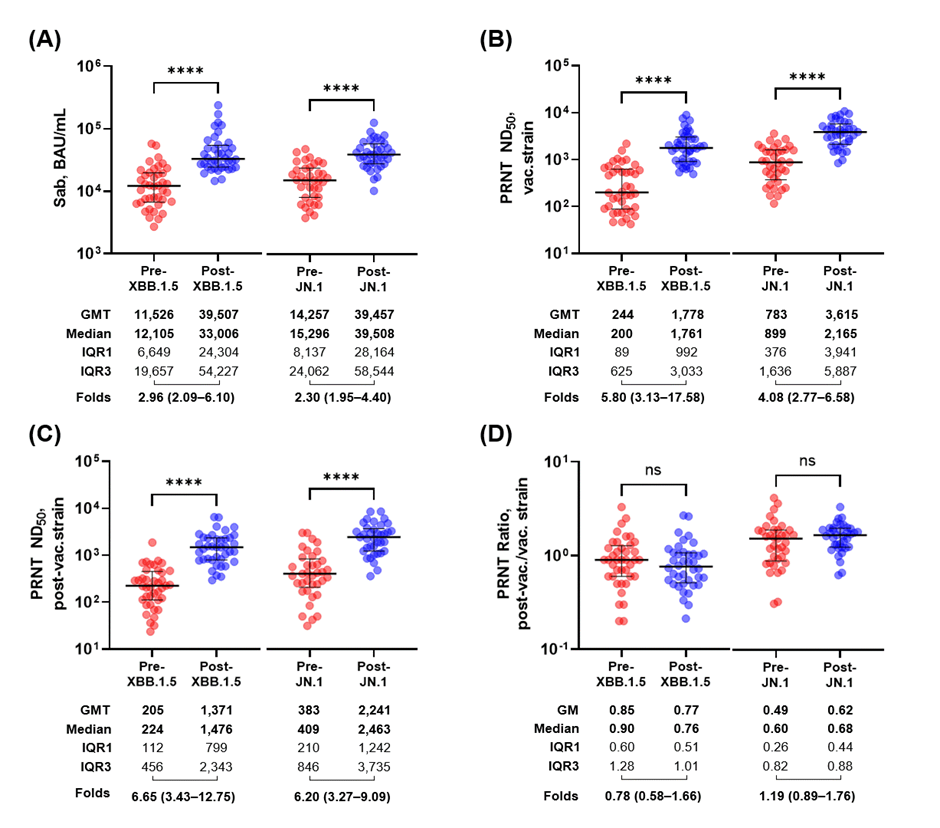


**Supplementary Figure 4.** **Humoral immune response to the XBB.1.5 and JN.1 MoVs.** Pre- and post-vaccination comparisons of the humoral immune responses to each vaccine are illustrated. Sab levels (A), PRNT titers against the vaccine strains (B), PRNT titers against the post-vaccine variants (C) and the PRNT ratio of the post-vaccine variant to the vaccine strain (D). *P* < 0.05 (*), *P* < 0.01 (**), *P* < 0.001 (***); and *P* < 0.0001 (****).

Abbreviations: MoV, monovalent vaccine; Sab, anti-spike antibody; PRNT, plaque-reduction neutralization test; ND_50_, 50% neutralization dose; GMT, geometric mean titer; IQR, interquartile range; GM, geometric mean
